# Altered reward processing in patients with lifelong premature ejaculation

**DOI:** 10.1038/s41598-023-44914-w

**Published:** 2023-10-16

**Authors:** Yansong Li, Xiaojun Li, Zixiang Wang, Xi Chen, Guillaume Sescousse, Pekka Santtila, Yutian Dai, Bing Zhang

**Affiliations:** 1 Department of Radiology, The Affiliated Drum Tower Hospital, Nanjing University Medical School, Nanjing, China; 2 Reward, Competition, and Social Neuroscience Lab, Department of Psychology, School of Social and Behavioral Sciences, Nanjing University, Nanjing, 210023 China; 3 School of Teacher Education, NanJing XiaoZhuang University, Nanjing, China; 4 Lyon Neuroscience Research Center—INSERM U1028—CNRS UMR5292, PSYR2 Team, University Lyon 1, Lyon, France; 5 Faculty of Arts and Sciences, New York University (NYU) Shanghai, Shanghai, China; 6 Department of Andrology, The Affiliated Drum Tower Hospital, Nanjing University Medical School, Nanjing, China; 7 Institute for Brain Sciences, Nanjing University, Nanjing, China

**Keywords:** Psychology, Sexual dysfunction

## Abstract

Given that sexual behavior is usually pleasurable and highly rewarding, it is surprising that there is as yet no known research to empirically assess how premature ejaculation (PE) patients respond to the rewarding aspect of sexual behavior. This study was designed to address this issue by evaluating how these men respond to the anticipation and hedonic experience of sexual rewards in comparison to non-sexual rewards. Thirty lifelong PE patients and thirty healthy controls (HCs) performed the incentive delay task manipulating both erotic and monetary rewards. Compared to HCs, lifelong PE patients exhibited significantly faster RTs to erotic cues than to monetary cues during reward anticipation. Meanwhile, hedonic experience ratings after obtaining the actual reward showed that erotic rewards were rated as more pleasant than monetary rewards only by lifelong PE patients, which was driven by a decreased sensitivity to experienced monetary rewards in lifelong PE patients compared to HCs. These findings indicate the existence of dysfunctional reward processing in lifelong PE patients, which is characterized by increased incentive motivation elicited by sexual cues and reduced hedonic impact of nonsexual rewards. This study may offer an insightful clue regarding how PE is related to the abnormal regulation of the rewarding aspect of sexual behavior.

## Introduction

Premature ejaculation (PE) refers to a type of male sexual dysfunction characterized by the inability to control and delay ejaculation during intercourse^1–3^. Most studies have reported its prevalence ranging from 12 to 30% in the general population^4^, and, consequently, it is recognized as the most common male sexual problem. PE has a detrimental influence on both sexual and relationship satisfaction^5^ and it is an important medical and social issue affecting the quality of life^6, 7^. To face this challenge, increasing research efforts have been devoted to understanding the etiology of PE^8^. However, our understanding of the exact pathogenetic mechanisms behind PE is still insufficient^9^.

Considering PE is a multidimensional medical problem that has both biological and psychological components^10^, elucidation of the psychological factors involved in PE would undoubtedly contribute to our understanding of the cause of the condition^11^. Furthermore, a correct understanding of these factors would potentially aid the identification of risk factors, the establishment of efficient diagnosis, and the development of more effective evidence-based treatments^12^. However, most previous work has used self-report measures to assess personality traits associated with susceptibility to PE and cognitions associated with sexual behavior in PE patients. These studies have found that PE patients report a larger range of negative psychosocial factors related to their sexual behavior, including sexual dissatisfaction, sexual frustration, personal distress, higher sexual anxiety, a lower feeling of control over sexual desires, and interpersonal difficulties with their partners^5, 13–19^. Despite important findings, the current literature fails to fully capture various aspects of the underlying psychopathologies of PE. Also, many of the reported correlates may be consequences of PE rather than etiological factors. Since the anticipation and experience of sexual intercourse are usually highly rewarding^20–22^, it seems reasonable to speculate that PE may be associated with the abnormal regulation of the rewarding properties of sexual behavior. In this sense, empirically assessing response to the rewarding aspect of sexual behavior in PE patients may help to deepen the insight into the underlying etiology of PE.

**Figure 1:**
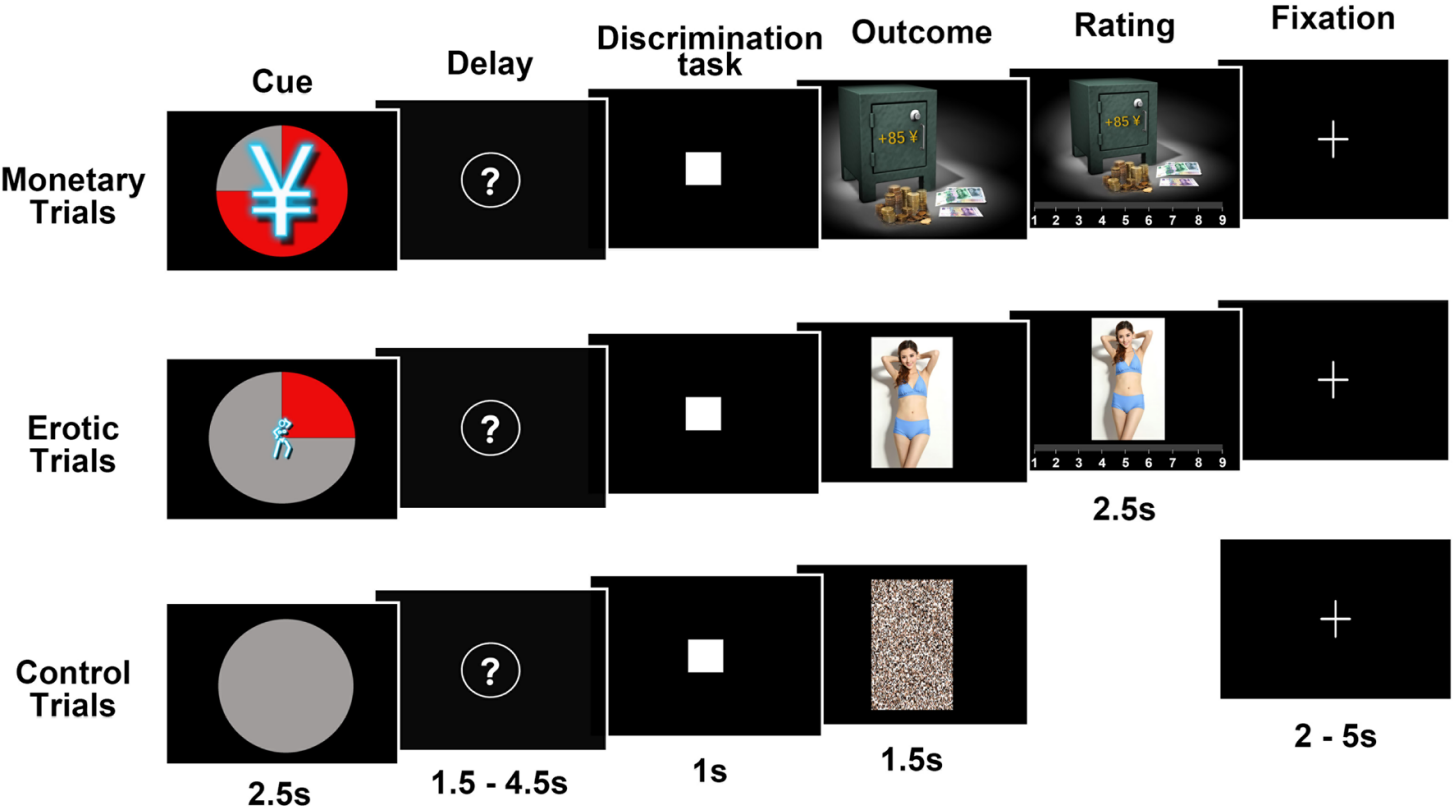
Incentive delay task. First, participants were presented with a cue signaling them information about the type (pictogram), intensity (size of pictogram), and probability (pie chart) of a possible reward. In the figure, we showed three cases: a 75% probability of getting a large amount of money (top), a 25% probability of receiving a low erotic content picture (middle), and a sure chance of getting nothing (control trials, bottom). Then, the cue was followed by a question mark, symbolizing a delay period during which a pseudorandom draw was conducted according to the announced probability. Following this anticipation phase, participants were required to perform a target discrimination task within 1 s. The target was either a triangle or a square. Both their performance and the result of the pseudorandom draw determined the nature of the outcome. In rewarded trials, participants received a monetary amount displayed on a safe (high or low amount, top) or an erotic picture (with high or low erotic content, middle), and had to provide a self-reported hedonic rating. In non-rewarded and control trials, subjects would get a scrambled picture (bottom).

Surprisingly, no work to date has attempted to address this issue in PE patients. Prior animal and human studies suggest dissociable psychological components of reward, which usually include incentive motivational processes elicited by reward-predicting cues during reward anticipation and hedonic processes (subjective pleasure) triggered by rewarding outcomes^23–26^. Based on these considerations, the present study was designed to empirically evaluate how lifelong PE patients respond to the reward properties of sexual activity in terms of how they respond to the anticipation and hedonic experience of sexual rewards in comparison to non-sexual rewards. We used an incentive delay task (IDT) involving both erotic and monetary rewards, which was described in our previous studies^27–29^. Specifically, the task included an anticipation phase where participants saw explicit cues that predicted either erotic or monetary rewards and a hedonic rating of the outcome phase where participants provided self-reported pleasantness ratings of these rewards (Fig. 1). Given the lack of previous empirical findings, we refrained from making specific hypotheses and considered this study to be exploratory.

## Results

### Accuracy

Our mixed ANOVA failed to reveal any significant effects (all *ps* > .05).

### Reaction times (RTs)

**Figure 2:**
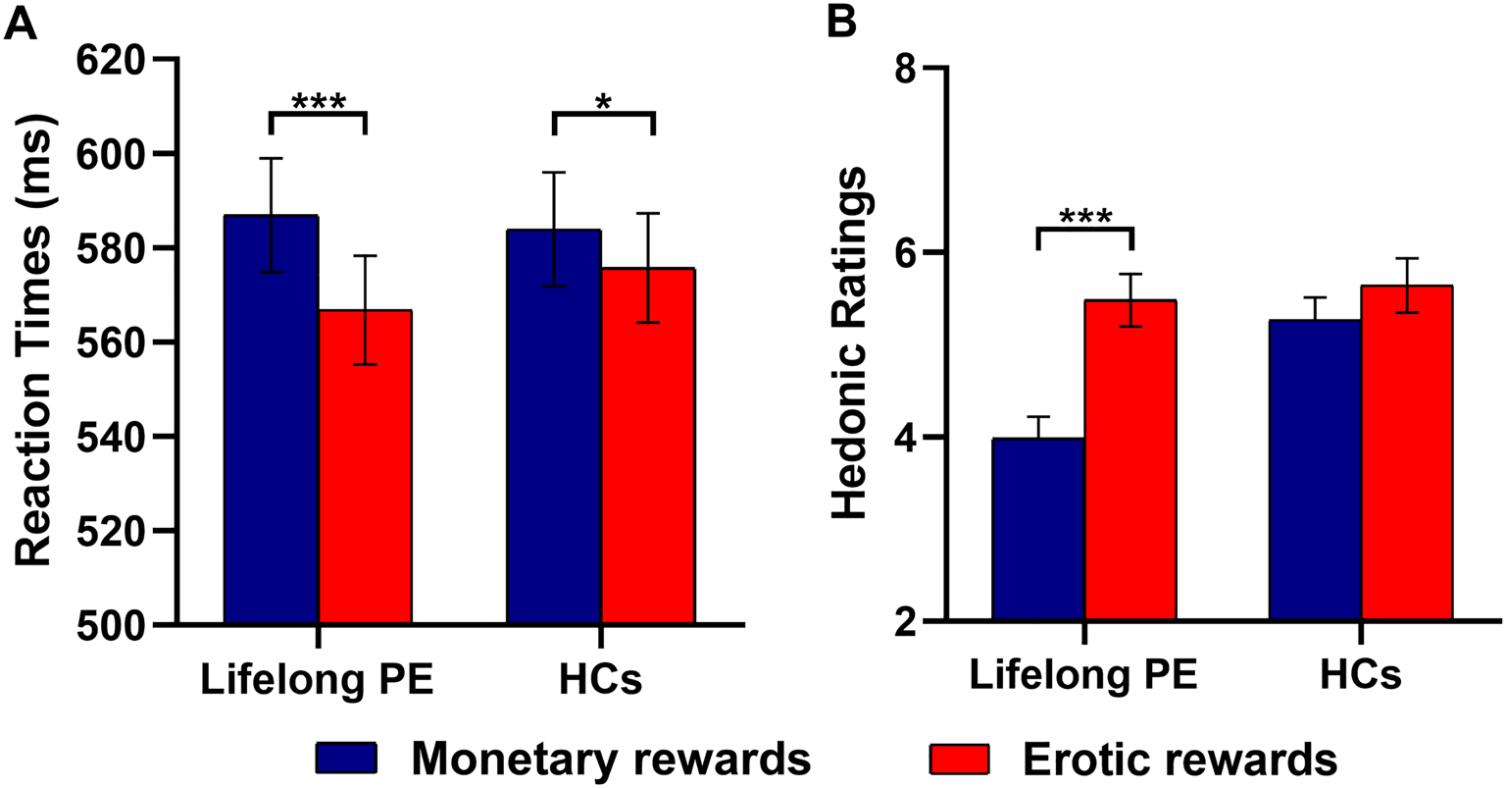
Behavioral results. (**A**) Reaction times (RTs) averaging over levels of reward intensity and reward probability as a function of reward type (monetary vs erotic) and group (lifelong PE patients vs HCs) in the discrimination task. Although RTs were significantly faster to erotic cues than to monetary cues in both lifelong PE patients (*p* < .001) and HCs (*p* < .05), the effect size was descriptively 79% smaller in in HCs than in lifelong PE patients. **(B**) Hedonic ratings averaging over levels of reward intensity and reward probability as a function of reward type (monetary/erotic) and group (lifelong PE patients vs HCs). A significant interaction between group and reward type was driven by higher hedonic ratings on erotic rewards than monetary rewards in Lifelong PE patients. Error bars indicate SD. ***p < .001, *p < .01.

Our mixed ANOVA on RTs revealed a significant main effect of reward type (F(1,58) = 27.95, *p* < .001, $\eta_{{\text{p}}}^{2}$ = 0.325) and a significant group × reward type interaction (F(1,58) = 4.99, *p* < .05, $\eta_{p}^{2}$ = 0.079) (Fig. 2A). An analysis of simple effects revealed that, although RTs were significantly faster for erotic cues than for monetary cues in both lifelong PE patients (erotic cues: M = 566.79 ms, SE = 11.54; monetary cues: M = 586.93 ms, SE = 12.11, *p* < .001, $\eta_{p}^{2}$ = 0.33) and HCs (erotic cues: M = 575.72 ms, SE = 11.54; monetary cues: M = 583.90 ms, SE = 12.11, *p* < .05, $\eta_{p}^{2}$ = 0.07), the effect size was descriptively 79% smaller in HCs than in lifelong PE patients. To identify whether such difference in effect sizes is indeed statistically significant, we performed further analysis to compare ΔRTs between lifelong PE patients and HCs. ΔRTs were calculated by subtracting RTs in monetary cued trials from RTs in erotic cued trials for each group. Our two-sample t-test revealed that ΔRTs were more pronounced in lifelong PE patients (M = − 20.13 ms, SE = 3.83) than in HCs (M = -8.18 ms, SE = 3.74) (t(58) = -2.23, p < .05, d = -0.58) (supplementary Fig. 1). There was also a significant main effect of reward intensity (F(1,58) = 8.51, *p* < .01, $\eta_{p}^{2}$ = 0.128), with RTs being faster on high-reward trials (M = 574.58 ms, SE = 8.34) than on low-reward trials (M = 582.09 ms, SD = 8.38, d = 0.90). Meanwhile, there was a significant main effect of reward probability (F(2,116) = 33.26, *p* < .001, $\eta_{p}^{2}$ = 0.364), with RTs being significantly faster for probabilities of 75% (M = 566.76 ms, SE = 8.05) than for probabilities of 50% (M = 580.81 ms, SE = 8.49, *p* < .05, d = 1.70) and 25% (M = 587.43 ms, SE = 8.62, *p* < .001, d = 2.48), and with RTs being significantly faster for probabilities of 50% than for probabilities of 25% (*p* < .001, d = 0.77). No other significant effects were found (all *ps* > .05).

### Hedonic ratings

Regarding hedonic ratings, our mixed ANOVA revealed a significant main effect of both group (F(1,54) = 4.98, *p* < .05, $\eta_{p}^{2}$ = 0.084) and reward type (F(1,58) = 24.16, *p* < .001, $\eta_{p}^{2}$ = 0.309). There was also a significant interaction between them (F(1,54) = 8.66, *p* < .01, $\eta_{p}^{2}$ = 0.138) (Fig. 2B). An analysis of simple effects revealed that lifelong PE patients rated erotic rewards (M = 5.48, SE = 0.28) significantly more pleasant than monetary rewards (M = 3.98, SE = 0.24, *p* < .001), while there was not a significant difference in the ratings of both types of rewards in HCs (erotic rewards: M = 5.64, SE = 0.29; monetary rewards: M = 5.27, SE = 0.24, *p* > .05). This finding seems to imply that lifelong PE patients process erotic rewards differently from healthy controls. However, given that the hedonic ratings of monetary rewards seem to be comparable across both groups, the significant difference in hedonic ratings between erotic and monetary rewards may result from reduced hedonic ratings of monetary rewards (nonsexual rewards) in lifelong PE patients compared to HCs. To promote our understanding of this issue, we further performed a simple effect analysis aiming at testing whether the effect of group on ratings depended on different levels of reward type. The results showed that lifelong PE patients rated monetary rewards (M = 3.98, SE = 0.24) significantly less pleasant than HCs (M = 5.27, SE = 0.24, *p* < .001), while there was not a significant difference in the hedonic ratings of erotic rewards across both groups (lifelong PE patients: M = 5.48, SE = 0.28; HCs: M = 5.64, SE = 0.29, *p* > .05) (supplementary Fig. 2). Moreover, there was a significant main effect of reward intensity (F(1,54) = 203.18, *p* < .001, $\eta_{p}^{2}$ = 0.790) and a significant interaction between reward intensity and reward type (F(1,54) = 31.37, *p* < .001, $\eta_{p}^{2}$ = 0.367). An analysis of simple effects revealed that pleasantness ratings for low monetary rewards (M = 2.87, SE = 0.18) were significantly lower than those for low erotic rewards (M = 4.48, SE = 0.24, *p* < .001), while those for high monetary rewards (M = 6.38, SE = 0.24) and for high erotic rewards (M = 6.65, SE = 0.22) were not significantly different (*p* > .05). In addition, there was a significant main effect of reward probability (F(2,108) = 6.35, *p* < .005, $\eta_{p}^{2}$ = 0.105) with pleasantness ratings for probabilities of 25% (M = 5.22, SE = 0.16) being significantly higher than those for probabilities of 75% (M = 4.97, SE = 0.17, *p* < .005, d = 1.51), and with pleasantness ratings for probabilities of 50% (M = 5.10, SE = 0.17) also being significantly higher than those for probabilities of 75% (*p* < .005, d = 0.73). Finally, no other significant effects were found (all *ps* > .05).

## Discussion

To the best of our knowledge, the present study is the first to empirically evaluate how lifelong PE patients respond to the anticipation and hedonic experience of sexual rewards in comparison to non-sexual rewards. Regarding reward anticipation, this study revealed that although the significant effect of reward type on RTs in HCs was found, lifelong PE patients exhibited significantly larger increased RT difference for erotic vs. monetary cues. Regarding hedonic experience ratings after obtaining the actual reward, only lifelong PE patients rated erotic rewards as more pleasant than monetary rewards. However, given that the hedonic ratings of monetary rewards seems to be comparable across both groups, our further analysis revealed that the significant difference in hedonic ratings between erotic and monetary rewards resulted from reduced hedonic ratings of monetary rewards (nonsexual rewards) in lifelong PE patients compared to HCs (supplementary Fig. 1). As such, the findings of this study may provide an insightful clue regarding how PE patients respond to the rewarding properties of sexual behavior and consequently shed light on the etiology of PE.

As pointed out above, reward-predicting cues are associated with incentive motivational processes^23–26^. Such incentive motivational properties of reward-related cues can serve to promote an approach toward and consumption of rewards and thus may bias behavioral choices toward potentially rewarding events. In this sense, our observation that relative to HCs, lifelong PE patients exhibited significantly larger increased RT difference for erotic vs. monetary cues implies an increase in incentive motivation to pursue erotic rewards compared to monetary rewards among them. Such hypersensitivity to erotic reward-predicting cues can in turn lead to excessive attribution of incentive salience to erotic reward-related representations, causing pathological ‘wanting’ to obtain erotic rewards.Sexual behavior is generally considered pleasurable and rewarding. It is not surprising hence that brain areas implicated in rewards are elicited when individuals anticipate such rewards. A wealth of evidence supports the notion that the connection of the ventral pallidum (VP) with the ventral striatum (VS), including the nucleus accumbens (NAcc), forms a subcortical neurocircuitry mediating incentive motivation elicited by reward-predicting cues^24, 26^. We suggest that the enhancing effect of PE on incentive motivation may arise from enhanced responsiveness of the VS-related motivational neurocircuitry toward erotic cues in lifelong PE patients. Considering that this subcortical incentive-motivational neurocircuitry is an important component of the mesolimbic dopamine system in humans^30^, we further postulate that these findings may reflect that dopaminergic (DA) hyperactivity is involved in PE. In this respect, our findings add to a growing literature supporting the argument that alterations in dopaminergic control of ejaculation may be an aetiological factor contributing to ejaculatory dysfunction including PE^31^. However, the majority of previous studies in animals and humans focus on the possible role of dopaminergic receptors or the dopamine transporter gene (DAT1) polymorphism and PE^31, 32^. Here, our observation during reward anticipation in lifelong PE patients seems to provide indirect evidence in support of the argument that the dysfunctional nigrostriatal dopamine pathway plays a special role in ejaculatory dysfunction including PE^31^, although this needs to be confirmed in studies that test this hypothesis directly. Meanwhile, the investigation of reward processing revealed dissociable psychological components of reward, which include not only incentive motivational processes elicited by reward-predicting cues during reward anticipation, but also hedonic processes (subjective pleasure) triggered by rewarding outcomes^23–26^. Thus, excessive attribution of incentive salience to erotic reward-related representations (relative to monetary rewards) may confer an imbalanced sensitivity to hedonic value of erotic and monetary rewards in lifelong PE patients. Given that such dissociable components of reward have been shown to be involved in distinct neural networks^33, 34^, PE may differentially affect incentive motivational processes and hedonic processes of reward processing. For the hedonic experience of actual rewards, we found that erotic rewards were rated as more pleasant than monetary rewards only in lifelong PE patients, which was driven by a reduced hedonic sensitivity to monetary (nonsexual) rewards. From this, it seems rather likely that lifelong PE patients may have allostatic changes in the hedonic set-point for monetary rewards and thus, attribute lower reward values to nonsexual rewards. Similar to our observation of the enhanced incentive motivational processes elicited by erotic cues in lifelong PE patients, we would assume that the reduced hedonic impact of obtained monetary rewards by PE patients may possibly reflect blunted responsiveness of the hedonic neurocircuitry that is implicated in subjective hedonic processing in humans. The prefrontal cortex mainly including the orbitofrontal cortex (OFC) is found to be central to the hedonic neurocircuitry, which is also a key component of the mesolimbic dopamine system in humans^35^. In this sense, it seems to be rational to speculate that PE can also reduce the hedonic impact of obtained monetary rewards, possibly via effects on the hedonic circuit of the mesolimbic dopamine system. Taken together, lifelong PE patients exhibited an allostatic shift in both the incentive motivational process of erotic reward processing and the hedonic/pleasure process of monetary reward processing. Given that hypersensitivity/hyposensitivity to reward has been argued to be one of two independent dimensions of impulsivity^36^, these findings likely reflect a changing balance in cognitive control capacities versus rewarding properties of sexual behavior in lifelong PE patients. Hence, excessive rewarding properties of sexual behavior may override cognitive control capacities in PE, which has recently been claimed to be a core mechanism underlying the aetiologies of PE^37^. In this sense, our findings may reveal new perspectives on the treatment options for PE. Future therapies for PE may include electrical stimulation or drugs that can interfere with incentive motivation and the hedonic experience of rewarding properties of sexual behaviors.

Finally, it is noted that behavioral measures of responses to standardized psychological tasks may provide a distinct, yet complementary approach to comprehensively evaluating the psychological functions of PE patients, when considering that self-report and behavioral measures have recently been claimed to tap very different response processes^38^. For this reason, this complementary approach may help to deepen the insight into the etiology of PE. Despite encouragement, it has not been until recently that researchers have begun to contribute to this understudied area of research. For example, a recent study provided an important clue into characterizing social cognitions in PE patients by showing a deficit in affective Theory of Mind (ToM) abilities of lifelong PE patients using an emotional intention recognition task^39^. This highlights a need to further advance our knowledge of the underlying psychopathologies of PE through this complementary approach.

## Limitations

In spite of our interesting findings, the present study includes limitations that warrant mention. First, the present study only included patients suffering from lifelong PE. Given that PE is a heterogeneous and multifactorial condition and the difference between PE subtypes has been increasingly highlighted^19, 40, 41^, this may affect the “generalizability” of our results to PE patients in general. Future research should aim at characterizing how different subforms of this condition respond to rewarding properties of sexual behavior. Second, given that the present study focused on the Incentive Delay Task which is a frequent measurement of reward processing, it is an open question to what extent the present results can be generalized into other types of paradigms measuring reward processing. As a consequence, it is necessary to incorporate different paradigms to better characterize how PE impacts the regulation of the rewarding aspect of sexual behavior in future studies. Third, since the coronavirus (COVID-19) pandemic significantly disrupted participants’ participation in our present study, this study involved a relatively small sample. Replications with larger samples would be welcome. Despite these limitations, we believe that our findings open up the possibility to gain further insights into functional characteristics and pathological signatures underlying sexual reward processing in PE patients.

## Conclusions

The present study was designed to characterize how lifelong PE patients respond to the rewarding properties of erotic stimuli. We used an incentive delay task involving both erotic and monetary rewards (money). By using this task, we were able to address how PE patients respond to the anticipation and hedonic experience of erotic rewards in comparison to monetary rewards. Our main finding was that compared to HCs, lifelong PE patients displayed significantly larger increased RT difference for erotic vs. monetary cues, indicating an enhancement of incentive motivation to pursue sexual rewards compared to nonsexual rewards in lifelong PE patients. In contrast, for hedonic experience ratings after obtaining the actual reward, erotic rewards were rated as more pleasant than monetary rewards only in lifelong PE patients, which was driven by a reduced hedonic sensitivity to monetary (nonsexual) rewards, thereby indicating PE can weaken the hedonic impact of obtained nonsexual rewards. These findings indicate the existence of a difference in reward processing in lifelong PE patients, which is characterized by an allostatic shift in both the incentive motivational process and the hedonic/pleasure process of reward processing. Therefore, the present study offers clues regarding how PE patients respond to the rewarding aspect of sexual behavior, and hence our findings may also have important implications for stimulating future research characterizing alterations in processing the rewarding aspect of sexual behavior in PE patients to optimize putative clinical interventions.

## Materials and methods

### Participants

The procedure for recruiting participants was similar to that described in our recent studies^42, 43^. We recruited 30 lifelong PE patients at the andrology outpatient clinic of Drum Tower Hospital, Affiliated Hospital of Nanjing University, and 30 age-matched healthy controls (HCs). All participants were right-handed and volunteered to participate in the present study. Moreover, consistent with previous work^44, 45^, to ensure a comparable state of motivation towards monetary stimuli, the two groups were matched in relation to income level. Written informed consent was obtained from all participants prior to participation and the Ethics Committee of the Drum Tower Hospital approved the study protocol. Written informed consent was also obtained from all participants for the publication of identifying information/images. This study has been performed in accordance with the ethical standards laid down in the 1964 Declaration of Helsinki and its later amendments.

All lifelong PE patients were diagnosed by our trained andrologist (Y.D.). The inclusion criteria for lifelong PE patients were: (1) fulfilling the diagnosed criteria for Lifelong PE according to the International Society for Sexual Medicine (ISSM) LPE Guidelines^46, 47^; (2) Intravaginal Ejaculation Latency Time (IELT) < 1 min; 3) Premature Ejaculation Diagnostic Tool (PEDT) scores > 11 (range 11–20)^48^; (3) International Index of Erectile Function-5 (IIEF-5) scores > 21^49^; (4) had had a stable, heterosexual relationship for at least 6 months. Meanwhile, the following exclusion criteria were used: (1) penile prosthesis or penile anatomical disorders; (2) a current or past history of substance dependence; (3) neurological disorders (i.e., epilepsy, neuromuscular disorders); (4) mental retardation; (5) any history of serious psychiatric disorders. Demographic and clinical characteristics are described in Table 1.

**Table 1:** Demographic and clinical characteristics of Lifelong PE patients and HCs (M ± SD).

	HCs (N = 30)	Lifelong PE patients (N = 30)	Group comparison
Age	27.60 ± 5.97	27.50 ± 3.18	t(58) = 0.08
Monthly income (¥)	5958.33 ± 2571.33	5003.33 ± 3401.87	t(58) = 1.23
IELT (mins)	15.48 ± 11.16	0.80 ± 0.67	t(58) = 7.19***
PEDT	3.73 ± 2.57	14.43 ± 3.23	t(58) = 14.18***
IIEF-5	23.27 ± 1.11	22.82 ± 0.92	t(58) = 1.66

IELT, Intravaginal Ejaculatory Latency Time; PEDT, Premature Ejaculation Diagnostic Tool; IIEF-5, International Index of Erectile Function-5; HCs, healthy controls; Lifelong PE patients, lifelong premature ejaculation patients; M, mean; SD, standard deviation. ***p < .001.

### Incentive delay task

We used the same task procedure described in our previous studies^27–29^. The task was performed in the Experimental Laboratory at the Department of Psychology, University of Nanjing. Participants were seated in a dimly light room facing a computer monitor placed at 80 cm distance from their eyes. The task was presented in E-Prime (Version 2.0, Psychology Software Tools Inc., Pittsburgh PA, USA). Participants were instructed that they were taking part in the task designed to assess their responses to the anticipation and receipt of rewarding stimuli. This incentive delay task (IDT) involved both erotic (sexual) and monetary rewards (money). Each trial of the task included an anticipation phase, a discrimination task, an outcome phase, and a hedonic rating phase (Fig. 1). During the anticipation phase, participants saw 1 of 12 explicit cues informing them about the type (monetary/erotic), probability (25%/50%/75%), and intensity (low/high) of an upcoming reward (2.5 s). In addition, a control cue was also included, which was related to a null reward probability. After a variable delay period (1.5–4.5 s), participants were instructed to perform a target discrimination task (a triangle or a square) as quickly as possible within a maximum time of 1 s with left and right index-finger button-press on a keyboard. Participants’ performance on this task would determine how the outcome was delivered. That is, correct responses on the task resulted in the delivery of the outcome of the pseudorandom draw that depended on the announced probability of the preceding cue, whereas erroneous or slow responses led to the omission of the outcome. In rewarded trials, outcomes would be either an erotic image or an amount of money displayed on a safe (1.5 s), whose intensity was either high or low depending on the preceding cue. Following each reward outcome, participants were instructed to provide a hedonic rating on a 1–9 continuous scale (1 = very little pleased; 9 = very highly pleased) (2.5 s). In both non-rewarded and control trials, participants were presented with ‘scrambled’ pictures. A fixation cross was finally employed resulting in an intertrial interval of variable length (2–5 s). Totally, there were 176 trials divided into 4 experimental blocks (57 trials each).

To make this task more appropriate for the Chinese participants, we made two minor modifications to it. One modification related to the actual numbers used in the monetary gains. We used the Chinese currency (¥) to denote the varying amounts of monetary reward at stake: the low amounts were ¥10, ¥15, or ¥20 and the high amounts were ¥80, ¥85, or ¥90. The other modification is related to erotic pictures. We selected high- and low-intensity erotic pictures from a recently released Chinese erotic picture dataset^50^. Similar to our previous work, the low-intensity erotic pictures displayed Chinese females in underwear or bathing suits, while the high-intensity pictures displayed naked females in an inviting posture.

### Statistical analysis

Independent sample t-tests were employed to examine between-group differences in age, income, IELT, PEDT, IIEF-5, SAI, BDI, and SAS. For accuracy and reaction times, obtained at the time of the discrimination task, as well as hedonic ratings, obtained at the time of outcome, three separate mixed analyses of variance (ANOVA) were performed, with group as a between-participants factor (lifelong PE patients versus HCs) and reward type (erotic versus monetary rewards), reward intensity (high versus low), and reward probability (25% versus 50% versus 75%) as within-participants factors.

All data were analyzed using IBM SPSS 23.0 (IBM Corp., Armonk, NY, USA). Statistical comparisons were made at *p* values of *p* < .05, with the Greenhouse–Geisser correction when violations of sphericity occurred.

### Supplementary Information

Supplementary Figures.

## Data Availability

The datasets used and/or analyzed during the current study are available from the corresponding author on reasonable request.
