# Supplementary material for: Altered reward processing in patients with lifelong premature ejaculation
Source: Sci Rep. 2023 Oct 16;13:17539. doi: 10.1038/s41598-023-44914-w (PMC10579392; doi:10.1038/s41598-023-44914-w)
Supplement: Supplementary file 1 — Supplementary Figures. [file 41598_2023_44914_MOESM1_ESM.docx]

**Supplementary materials**

**Altered reward processing in patients with lifelong premature ejaculation**

Yansong Li^1,2,7*#^, Xiaojun Li^3*^, Zixiang Wang^2^**,** Xi Chen^2^**,** Guillaume Sescousse^4^, Pekka Santtila^5^, Yutian Dai^6#^, Bing Zhang^1#^

^1^ Department of Radiology, The Affiliated Drum Tower Hospital of Nanjing University Medical School, Nanjing, China

^2^ Reward, Competition, and Social Neuroscience Lab, Department of Psychology, School of Social and Behavioral Sciences, Nanjing University, Nanjing, China

^3^ School of Teacher Education, NanJing XiaoZhuang University, Nanjing, China

^4^ Lyon Neuroscience Research Center—INSERM U1028—CNRS UMR5292, PSYR2 Team, University Lyon 1, Lyon, France

^5^ Faculty of Arts and Sciences, New York University (NYU) Shanghai, Shanghai, China

^6^ Department of Andrology, The Affiliated Drum Tower Hospital of Nanjing University Medical School, Nanjing, China

^7^ Institute for Brain Sciences, Nanjing University, Nanjing, China

**
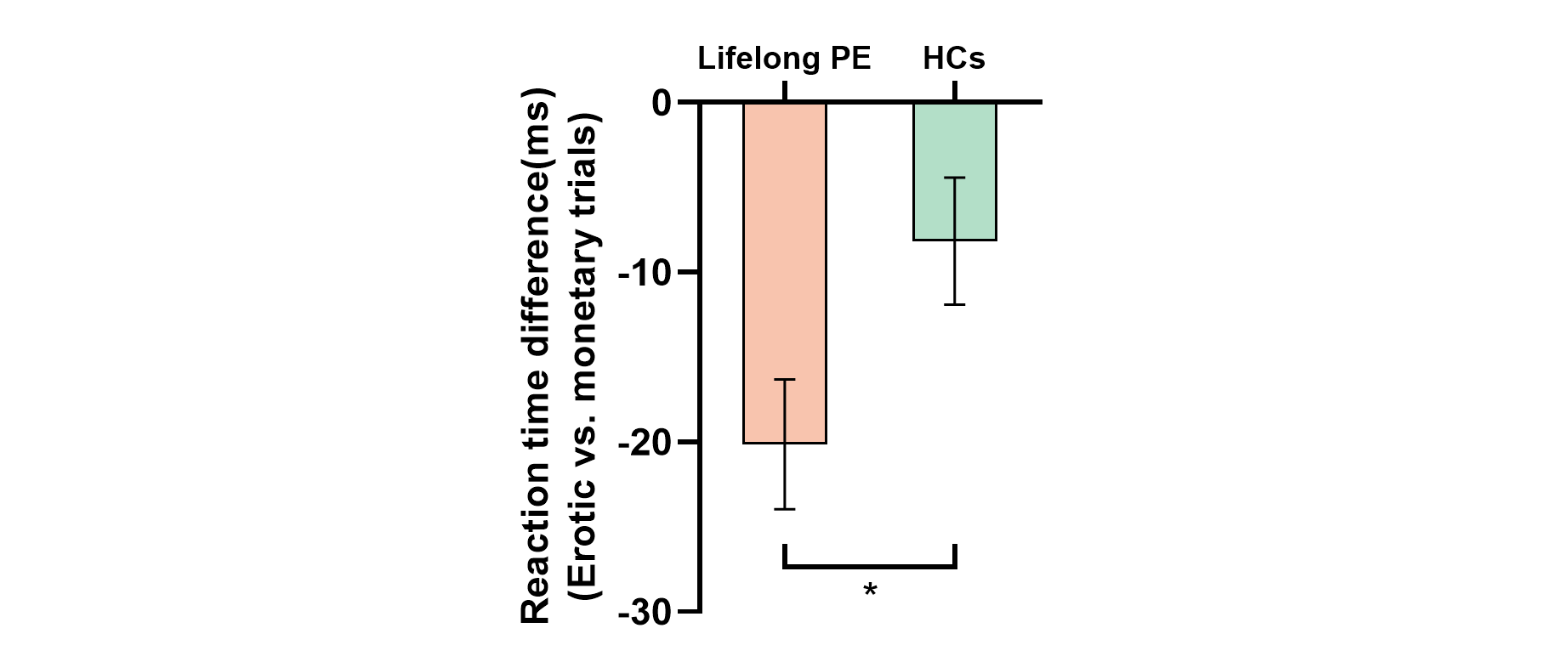
**

**Supplementary Figure 1. Reaction time difference of erotic vs. monetary cued trials as a function of group (lifelong PE patients vs HCs)**. The reaction time difference was more pronounced in lifelong PE patients than in HCs. Error bars indicate SE. * *p* < .05.

**
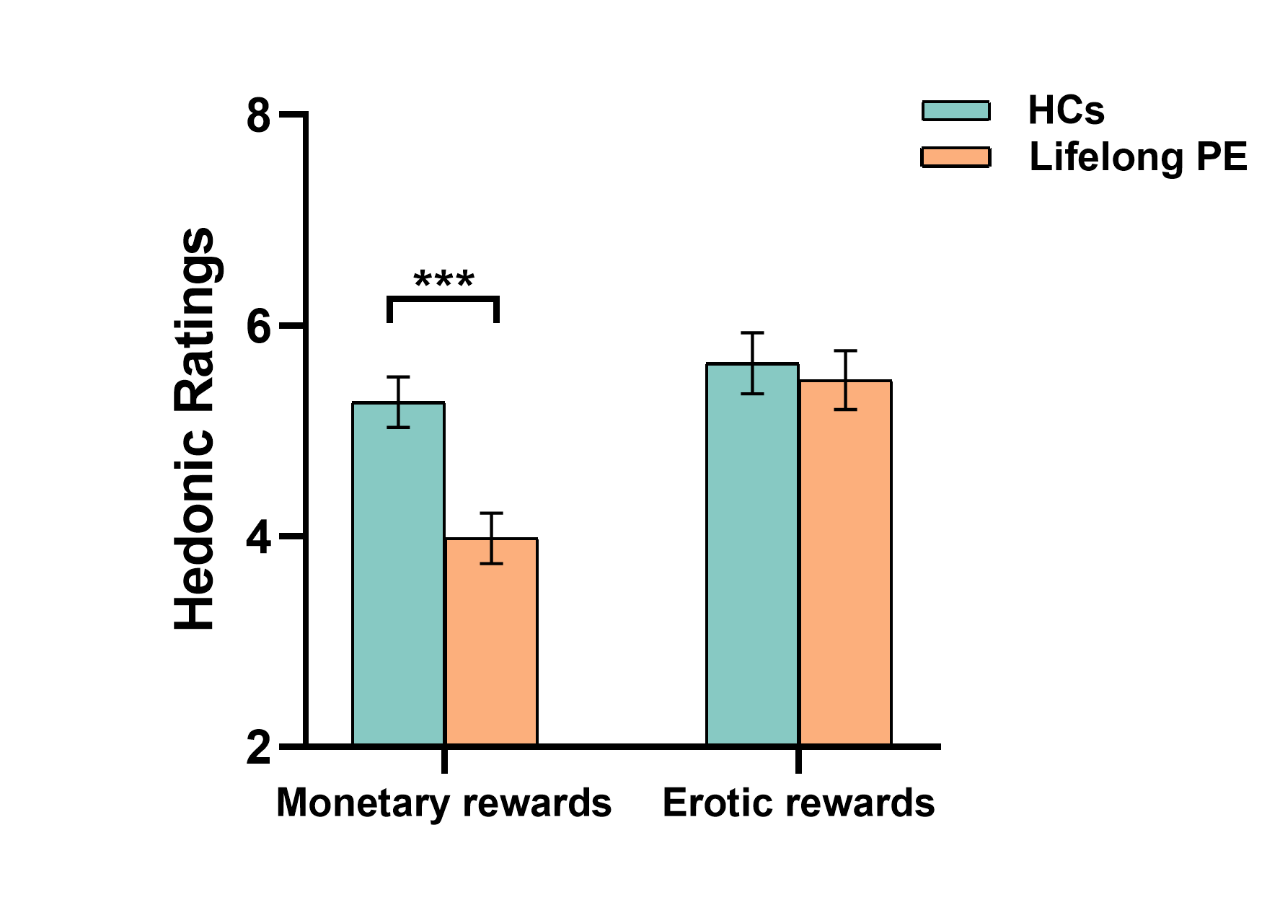
**

**Supplementary Figure 2. Hedonic ratings as a function of reward type (monetary/erotic) and group (lifelong PE patients vs HCs).** A significant interaction between group and reward type was driven by lower hedonic ratings on monetary rewards in lifelong PE patients than in HCs. Error bars indicate SE. *** *p* < .001.
